# Neurodevelopmental retardation and neurological symptoms in homozygous variegate porphyria: two new cases and a literature review

**DOI:** 10.1186/s13023-025-03606-6

**Published:** 2025-03-20

**Authors:** Nadja Kaiser, Janine Magg, Thomas Nägele, Nicole Wolf, Ingeborg Krägeloh-Mann

**Affiliations:** 1 Department of Neuropaediatrics, Developmental Neurology, Social Paediatrics, University Children’s Hospital, Tübingen, Germany; 2 Department of Radiology, Section of Diagnostic and Interventional Neuroradiology, University Hospital Tübingen, Tübingen, Germany; 3 Department of Child Neurology, Emma Children’s Hospital, Amsterdam UMC - Locatie VUMC and Amsterdam Neuroscience, Vrije Universiteit, Amsterdam, The Netherlands

**Keywords:** Developmental delay, Childhood epilepsy, Hypomyelination, Skin lesions, Variegate porphyria

## Abstract

**Background:**

Genetic porphyrias, namely in their homozygous form, may cause a neurodevelopmental disorder which may even be the clinically dominant feature. But few cases have been described so far. The majority of neurodevelopmental disorders has a genetic cause and there is a big overlap of the clinical presentations due to unspecific symptoms. Additional specific clinical symptoms may enable a phenotypically orientated biochemical and genetic diagnostic approach. Skin lesions occurring in the neonatal period or the first years of life in a child with developmental delay may hint at a genetic porphyria.

**Methods:**

We describe the clinical features, biochemical and genetic findings in two new cases, sister and brother, of biallelic resp. homozygous variegate porphyria and review all case reports published until December 2023 after systematic searches in PubMed, MEDLINE, Cochrane and Web of Science.

**Results:**

A total of 19 patients with biallelic, largely homozygous variegate porphyria have so far been reported of whom 16 were confirmed by genetic testing. In 11 patients, neurodevelopmental problems were reported in addition to skin lesions. Additional symptoms were nystagmus, epileptic seizures as well as sensory neuropathy. Only 2 patients received a brain MRI showing a severe deficit of myelination at the age of 2–3 years suggesting that neurodevelopmental delay in HVP may be associated to hypomyelination. This article adds two cases of a genetic porphyria with developmental delay and epilepsy as well as skin lesions. In our two cases biochemistry revealed a porphyria and consecutive molecular genetic testing showed in each case a homozygous variant in the *PPOX* gene, which corresponds to a variegate porphyria. Interestingly, magnetic resonance imaging of the brain revealed a severe myelin deficit suggesting hypomyelination in both children.

**Conclusions:**

In children with a developmental disorder of unknown cause and early childhood epilepsy, an abnormally light-sensitive or fragile skin may indicate a primary genetic porphyria. Especially variegate porphyria with biallelic variants may present as neurodevelopmental disorder with hypomyelination.

## Background

Variegate porphyria (VP, OMIM #176200) is one of eight genetic porphyrias. Clinically it is characterized by cutaneous manifestations and, especially in phases of exacerbation, by neurovisceral symptoms [13]. VP is a metabolic disease due to pathogenic variants in the protoporphyrinogen oxidase (PPOX) gene which leads to a strongly decreased activity of protoporphyrinogen oxidase, the penultimate enzyme in the hembiosynthesis pathway [13, 24]. Most patients with autosomal dominantly inherited VP are genetically heterozygous for a pathogenic variant in the *PPOX* gene [22]. As heterozygous variegate porphyria shows an incomplete penetrance of approximately 40%, many carriers may be asymptomatic during their whole life [12]. If symptomatic, heterozygous VP usually presents in adulthood and rarely before puberty [7, 22], with a combination of severe skin disease and acute symptoms with attacks of severe abdominal pain, neurologic symptoms including peripheral nerve palsies, seizures and nausea as well as nonspecific psychiatric abnormalities.

On the other hand, rare cases of so called homozygous variegate porphyria (HVP) which include biallelic pathogenic variants in homozygous and compound heterozygous form have been described with disease onset already in infancy [27, 31]. Usually the affected children became symptomatic in the first days or months of life. Heterozygous and homozygous cases do not only differ concerning the age at onset, but also regarding the clinical presentation. Most of the HVP cases lead to a variable neurodevelopmental disorder while acute neurovisceral attacks have not been reported. With the description of two new cases and a review of the literature, we want to draw attention to the fact that genetic porphyrias, namely in their homozygous form, may present as a predominantly neurodevelopmental disorder, a fact which paediatric neurologists may not be aware of.

## Methods

All cases of homozygous variegate porphyria reported until December 2023 were collected by conducting systematic searches in PubMed, MEDLINE, Cochrane and Web of Science using the search term “homozygous variegate porphyria”. The search identified 29 publications, which report a total of 19 patients with HPV. Data of patients that were reported repeatedly were summarized and interpreted accordingly. The data and information gathered and reviewed from all reports include the basic patient data (gender, age of onset, age at last clinical report), clinical features regarding dermatological, neurological and other symptoms as well as biochemical and genetic results and information about brain imaging. Additionally, the clinical, biochemical and genetic data of two new cases of HVP are presented.

## Results

### Review of the available literature about cases of homozygous variegate porphyria

The literature search on “homozygous variegate porphyria” yielded 29 publications in total (Table 2). Review of all so far reported cases of so-called homozygous variegate porphyria (HVP), comprising biallelic homozygous and compound heterozygous variants, resulted in a total of 19 reported patients. Due to missing genetic confirmation 3 patients [4, 6, 19] were excluded from workup in this review. Genetic confirmation of the clinical and/or biochemical diagnosis was established in 15 patients (94%) of whom 7 were carrying a homozygous and 8 a compound heterozygous mutation. A sibling of one of these patients was included with the corresponding clinical symptoms as his brother, although no genetic testing was done [16]. The summary of the literature is listed in Table 2.

**Table 1:** Biochemical analyses of two new HPV cases

Sample	Metabolite (unit)	Patient 1 (at the age of 10 d)	Patient 2 (at the age of 6 ½ y)	Reference value
Blood	Soluble protoporphyrin in erythrocytes (nmol/l)	1583	1256	9–89
	Total protoporphyrin in erythrocytes (nmol/l)	4176	4374	< 500
Urine	Coproporphyrin/creatinine (µg/g)	Not quantifiable	3 277	< 120
	Uroporphyrin/creatinine (µg/g)	Not quantifiable	100	< 33
	Porphobilinogen/creatinine (µmol/g)	48	15	< 8
	5-aminolevulinacid (5-ALA)/creatinine (µmol/g)	92	71.7	< 25
	Total porphyrin/creatinine (µg/g)	34 128	3 477	< 174
Faeces	Pentacarboxyporphyrin (µg/g)	104.2	Not obtained	< 3
	Coproporphyrin (µg/g])	65.9	Not obtained	< 24
	Total porphyrin (µg/g)	334	Not obtained	< 85

Urine, blood and faeces were analyzed in patient 1, urine and blood in patient 2. Both patients had highly elevated porphyrin levels in blood indicating a porphyria and very high protoporphyrin levels in erythrocytes. In patient 1, biochemical differentiation was complicated due to partially non-quantifiable values. In patient 2, biochemical analysis revealed a predominance of coproporphyrin. *Reference values for porphyrin/creatinine quotients are established only for adults*

**Table 2:** Summary of all previously reported HVP cases

References	Sex	Age at onset	Age at last clinical report	Neurological symptoms	Cutaneous symptoms	Other symptoms	Biochemistry consistent with VP	cMRI	Genetics
[32]	M	2 y	7 y	Seizures, delayed developmental milestones, learning disability, tremor	Blisters, hyperpigmentation, photosensitivity	Brachydactyly, short stature, aggressive behavior	n. i.	Severely delayed myelination	Homozygous, c.1072G > A, p.G358R
[1]	F	2 m	3 y	Seizures, nystagmus, developmental delay	Blisters, photosensitivity (at 6 m)	Brachydactyly	Yes	n. i.	Homozygous, p.Glu339Lys
[1]	M	n. i.	16 y	Seizures, severe developmental delay	Severe photosensitivity, multiple wounds	Amputation of fingers	Yes	n. i.	Homozygous, p.Glu339Lys
[29]	M	n. i.	n. i.	n. i.	Hyperpigmentation, blisters, photosensitivity	Brachydactyly	n. i.	n. i.	Homozygous, c.502C > T; (p.Arg168Cys)
[29]	M	n. i.	n. i.	n. i.	Hyperpigmentation, blisters, photosensitivity	Brachydactyly	n. i.	n. i.	Homozygous, c.502C > T; (p.Arg168Cys)
[25]	M	2 m	4 y	Nystagmus (at 2 m), developmental delay, truncal ataxia, intention tremor, microcephalia	Photosensitivity (at 2 y)	Brachydactyly	Yes	Hypo-myelination (at 18 + 24 month)	Compound heterozygous, c.169G > C (p.Gly57Arg)/c.1259C > G (p.Pro420Arg)
[26]	M	6 m	7 y	No	Severe photosensitivity (at 6 m)	Brachydactyly, growth retardation, short stature	Yes	n. i.	Compound heterozygous, G232S/ 1330delCT
[5]	F	19 y	26 y	No	Photomutilitation of the fingers	19 y: acute porphyric crisis	Yes	n. i.	Compound heterozygous, R59W/R138P
[5]	F	10 m	7 y	Nystagmus, sensory neuropathy, poor concentration	Photosensitivity (at 10 m)	brachydactyly, myopia	Yes	n. i.	Compound heterozygous, R59W/Y348C
[28]	M	8 m	16 y	No	Skin lesions	Clinodactyly	n. i.	n. i.	Homozygous, c.1046A > C (D349A)
[11] (clinical), [28] (genetics)	M	5 m	16 y	Seizures (at 5 m), developmental delay, nystagmus, intellectual disability	Skin lesions, blistering	Growth retardation, clinodactyly	Yes	n. i.	Homozygous, c.1297G > C (A433P)
[20] (clinical), [14] (genetics)	M	< 1 m	5 y	Ssensory polyneuropathy, mild developmental delay	Photosensitivity, severe bullous skin disease (first days of life)	Brachydactyly, IgA nephropathy, increased intraocular pressure	Yes	n. i.	Compound heterozygous, c.35T > C (I12T)/c.767C > G (P256R)
[19] (clinical), [28] (genetics)	F	9 m	33 y	One febrile convulsion (at 3—4 y), normal intelligence	Photosensitivity (at 9 m)	Short stature, brachydactyly, clinodactyly	Yes	No	Compound heterozygous, c.657–658 ins AAGGCCAGCGCC (A219KANA)/vs 11-11T > G (mutIntron11) **
[16] (clinical), [28] (genetics)	M	< 1 m	6 y	Nystagmus (at 2 m), convulsions (at 4 m), intellectual disability	Severe skin lesions (first days of life)	Growth retardation, clinodactyly	Yes	n. i.	Compound heterozygous, c.1072G > A (G358R)/ IVS7 + 1 del 18 bp (SD mutdelEx7)
[16]	F	< 1 m	8 m	Mild nystagmus (at 3 weeks), developmental delay (at 8 m), convulsions	Severe skin lesions (first days of life)	n. i.	Yes	n. i.	n. i. (sibling of child above)
[21] (clinical), [28] (genetics) *	F	18 m	14 y	Two convulsions during first y of life, intellectual disability	Photosensitivity, blisters, crusts and erosions (at 18 m)	Clinodactyly, normal growth	Yes	n. i.	Compound heterozygous, c.505G > A (G169E)/c.1072G > A (G358R)
New cases of HVP									
	F	1 day of life	7 y	Nystagmus, developmental delay, seizures	Blistering, photosensitivity, skin lesions	Hepatopathy in the neonatal period, short stature, microcephaly, joint contractures, brachydactyly	Yes	Hypomyelination	Homozygous c.164 > C (p.Glu55Ala)
	M	First years of life	14 y	Nystagmus, developmental delay, seizures	Skin lesions, photosensitivity	Short stature, microcephaly, joint contractures, brachydactyly	Yes	Hypomyelination	Homozygous c.164 > C (p.Glu55Ala)

DNA variants are represented as reported in the respective publication. M = male, F = female, m = month, y = year, n. i. = no information, *genetics also in McGrath et al. 1997 and Frank et al. 1998, **different results in Palmer 2001: c.657 ins 12 bp (A219KASA)/IVS 11-1G > A (mutIntron11)

The patients with biallelic *PPOX* variants were in general more severely affected than heterozygous patients. In addition to skin lesions most of them showed a varying combination of neurologic and/or neurodevelopmental symptoms including nystagmus, epileptic seizures, developmental delay, intellectual disability as well as sensory neuropathy. Additional repeatedly reported symptoms are growth retardation, brachy- and/or clinodactyly and myopia.

Out of the 16 cases with HVP, 12 patients (75%) had disease onset in the first two years of life, 10 of them (63%) even within the first 12 months, and 11 (69%) of the 16 patients showed neurological problems such as developmental delay/intellectual disability (9/11 or 82%) and/or seizures (8/11 or 73%). Nystagmus was reported in 6 of the 11 patients (55%). For 2 cases there was no information available concerning their general health. One patient showed—although classified as compound heterozygous—the typical aspects of a heterozygous VP (disease manifestation after adolescence with an acute porphyric crisis [5]. Especially in compound heterozygous patients, residual PPO activity of one of the variants might make a relevant difference regarding the clinical severity of the disease. For two patients from Pakistan with a confirmed homozygous mutation there is unfortunately nearly no clinical information [29].

In only two of the reviewed cases brain MRI was reported. In the first patient, at the age of 2 years the T2 weighted sequences showed complete absence of myelin including in the cerebellar white matter [25]. In the second case, severely delayed myelination at the age of 3 years was described [32].

Interestingly, to date no homozygous R59W variant carriers have been detected. The R59W DNA variant is the most prevalent in South Africa, supposedly due to a founder effect. Patients carrying the R59W variant in a heterozygous state are often severely affected, though there is no established genotype–phenotype correlation. Missing cases of homozygosity raise the question whether homozygosity for R59W variant is lethal.

### Two new cases of homozygous variegate porphyria

**Fig. 1:**
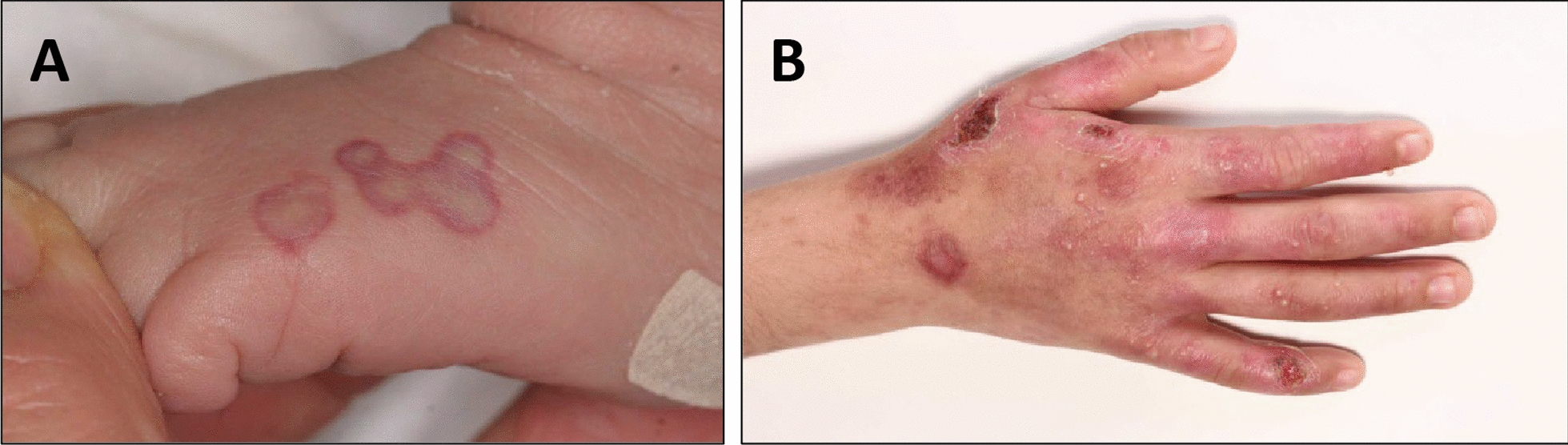
Skin lesions. **A** patient 1, first week of life: erythema after direct light exposure (pulse oximeter). **B** patient 2, at the age of 6 years: erosions and scars on sun exposed skin.

We identified two new cases of homozygous variegate porphyria that have so far not been published. Patient 1 is a now 7-year-old girl. She was admitted to our neonatal department because of hepatopathy on her first day of life, showing cutaneous lesions caused by the light of a pulse oximeter (Fig. 1A). Nystagmus became apparent at the age of 6 weeks. She developed epileptic seizures, mainly bilateral tonic seizures with eye blinking, at the age of 3 months which did not respond to levetiracetam, but to a combination of valproic acid and phenobarbital. Her subsequent development was globally delayed. Currently, she is able to walk without aid on level ground and expresses herself in simple two-word combinations. She attends a school for children with intellectual disability. She has short stature and microcephaly (at the age of 7 years: height 114 cm (P1, SDS: − 2.24), weight 19 kg (P2, SDS:− 2.02), BMI 14.6 kg/m^2^ (P25, SDS:− 0.68) and head circumference 44.7 cm (< P1, SDS: − 5.16), at birth: height 46 cm (< P1, SDS:− 3.02), weight 2450 g (< P1, SDS:− 2.99), head circumference 32 cm (< P1, SDS:− 2.53). She has serious skin lesions in the sun exposed areas, and as a result of scratching she is developing scars and contractures, leading to the development of brachydactyly. To date no acute neurovisceral attack has occurred.

Patient 2 is the brother of patient 1, a now 14-year-old boy who was born in Syria. The parents reported a primary global developmental delay. He was able to walk without aid at the age of 5 to 6 years, but still shows some deficits in gross and fine motor skills. He is able to talk in Arabic and German in sentences consisting of several words and he can count until 20. He also needs special education. He also has short stature and microcephaly (at the age of 14 years: height 145 cm (P1, SDS:− 2.40), weight 39 kg (P5, SDS:− 1.72), BMI 18.6 kg/m2 (P40, SDS:− 0.24) and head circumference 48.1 cm (< P1, SDS: − 4.32), anthropometric data at birth not available). The parents described a nystagmus in the first years of his childhood consistent with early medical reports. The nystagmus was no longer present when he was first seen at our institution at the age of 6 years. Generalized epileptic seizures were reported before the age of 6 months. At the presentation in our department he was seizure-free with valproic acid treatment, which was ended at the age of 7 years. Only one tonic–clonic seizure occurred in the 6 years after his first visit. At the age of 14 years he had two more generalized seizures which led again to treatment with valproic acid. He shows blistering, erosions, scars and pigment aberrations on the sun exposed skin of his face and hands (Fig. 1B). According to his parents, onset of skin lesions was in his third year of life. Now he is developing scars and contractures, with the appearance of brachydactyly. The neurodevelopmental symptoms and the fragile skin had been considered as independent disorders before the birth of his sister. Like his sister he has not experienced neurovisceral episodes so far. The consanguineous parents and two other siblings are not affected to date.

Diagnostic measures were biochemical analysis of blood and urine samples and genetic testing with sanger sequencing (patient 1 and 2) and whole exome sequencing (patient 2). Both patients underwent brain MRI. Informed consent of the parents prior to the biochemical and genetic testing was obtained. In patient 1 there was severe porphyrinuria with high total porphyrins, an increase in total faecal porphyrins as well as elevated protoporphyrins in plasma and erythrocytes and a positive fluorescence scan with an emission maximum at 624 nm (Table 1).

The clinical presentation and the biochemical findings led to the diagnosis of a porphyria in patient 1. Precise biochemical interpretation was difficult because of overlapping signals in the chromatographic analysis and missing reference values for newborns. Based on the early manifestation initially a harderoporphyria, which is the neonatal-onset form of homozygous hereditary coproporphyria [10], was discussed, but the molecular genetic analysis of the *CPOX* gene was normal. Because of the biochemical results, we then decided to analyze the *PPOX* gene which codes for protoporphyrinogen oxidase. Sanger sequencing revealed the homozygous variant c.164A > C (p.Glu55Ala) in exon 3, which has previously not been reported and was classified as a variant of uncertain significance (class 3) according to the *American College of Medical Genetics* guidelines. The biochemical analysis of the brother, patient 2, showed a significant porphyrinuria with dominance of coprophorphyrins and elevated plasma protoporphyrins as well as a positive fluorescence scan of blood plasma with an emission maximum at 626 nm confirming a porphyria. *PPOX* was also analyzed and the same homozygous variant as in patient 1 was found. This variant has not been described in the databases so far (ClinVar, gnomeAD), but in silico predictions in three out of four programs (MutationTaster, SIFT, PolyPhen) rated it as possibly pathogenic. In order to exclude other genetic causes for the global developmental delay patient 1 additionally underwent whole exome sequencing which confirmed the *PPOX* variant but did not reveal any other clinically relevant DNA variants based on the routine analysis tools at that time. Likewise, chromosomal analysis was normal and microarray analysis did not indicate a genomic imbalance.

**Fig. 2:**
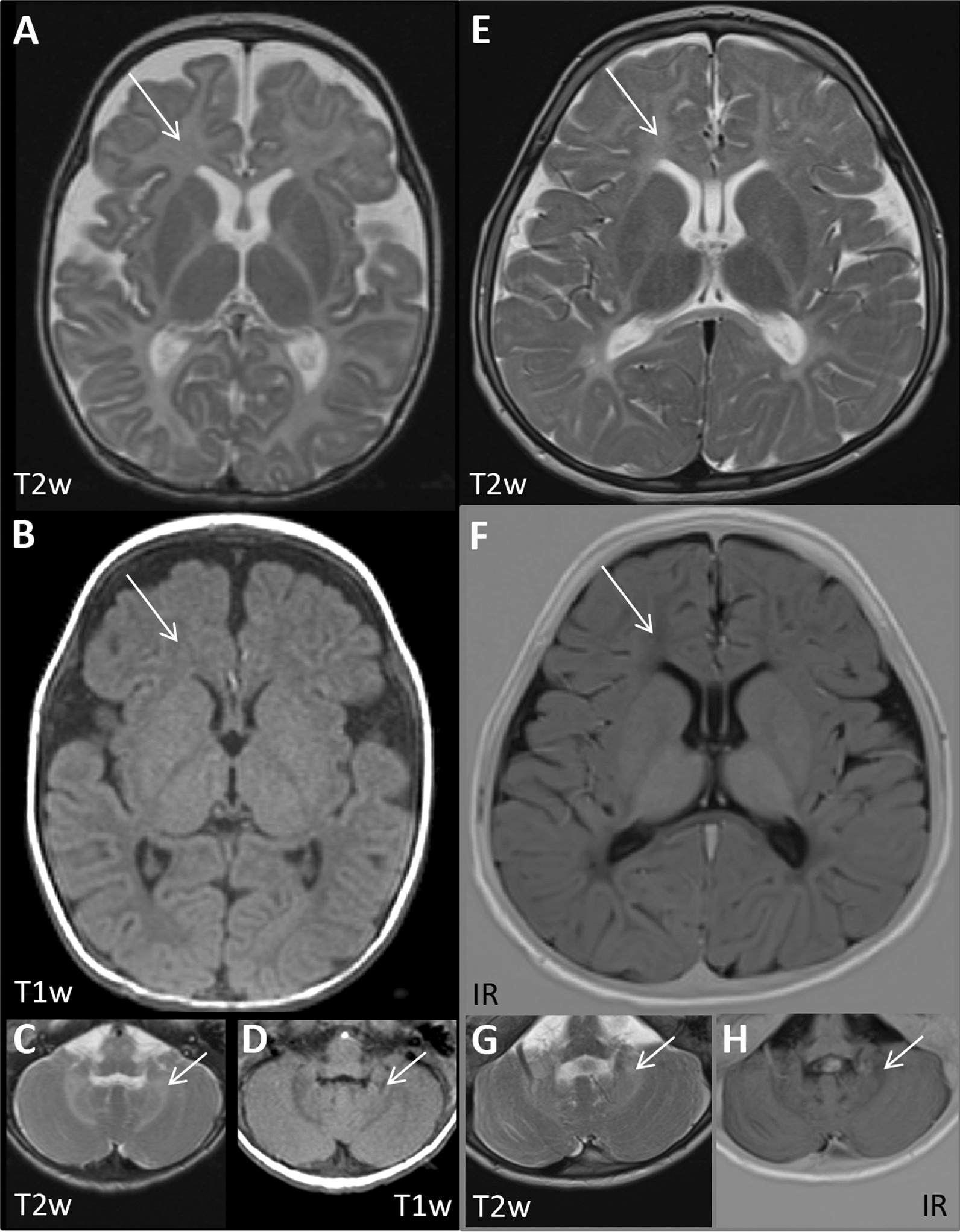
Brain MRIs of patient 1 at the age of 5 months **A**–**D** and patient 2 at the age of 3 years and 4 months **E**–**H** show hyperintensity of cerebral (arrows in A + B and E + F) and cerebellar (arrows in C + D and G + H) white matter on T2w axial images and hypointensity on T1w and IR images respectively, including the corpus callosum and the inner capsule which indicates the absence of regular myelination, suggesting severe hypomyelination as defined by a substantial and permanent deficit in CNS myelin deposition

MRI was obtained in both children at different ages. At the age of 5 months, the girl showed global hyperintensity of the cerebral as well as the cerebellar white matter on T2w images, whereas the signal of the white matter was hypointense on T1w images (Fig. 2A–D). At the age of 3 years and 4 months, the boy also showed hyperintensity of the cerebral and cerebellar white matter on T2w images including corpus callosum and the inner capsule, while on T1w and IR images these structures were hypointense (Fig. 2E–H). These findings indicate the absence of regular myelin deposition and suggest hypomyelination in both children as defined by a substantial and permanent deficit in CNS myelin deposition [30]. As there is virtually no myelin correlate in the cerebellar white matter and on T1w and IR images in the boy at an age when myelination should be complete, the hypomyelination seems to be severe. In sum, our patients presented clinically and biochemically with a porphyria and a global developmental delay as well as an epilepsy and a nystagmus. The results of the genetic testing suggested a homozygous variegate porphyria (HVP). The course of the disease is similar in both children.

### Summary and discussion

Our literature research revealed that most of the reported HVP patients showed, in addition to skin lesions, a varying combination of neurologic and/or neurodevelopmental symptoms. These include nystagmus, epileptic seizures, developmental delay, intellectual disability and sensory neuropathy. We therefore concluded that the homozygous *PPOX *variant in our two patients is also the cause of the neurologic symptoms. It may be argued that there is a high risk of other genetic causes for the global developmental delay as parents were consanguineous. But chromosomal analysis, array analysis and whole exome sequencing (patient 1) did not reveal any further genetic changes explaining the developmental delay. Genetic testing of the parents of our patients in order to confirm heterozygosity could not be performed so far. In the regular follow-up examinations both children showed continuous slow developmental progress within the limits of their intellectual disability. The findings of absent or severely delayed myelination in two earlier reported HVP patients as described above are in accordance with the MRI findings in our two cases. This indicates that severe hypomyelination is another feature in biallelic variegate porphyria, contributing to the neurodevelopmental disorder in these children. Why myelination is impaired, is unknown. Porphyrins lead to oxidative stress and their accumulation has been shown to lead to intermediate filament aggregation, damage to the endoplasmatic reticulum and disruption of protein turnover [17]. Oligodendrocyte precursors are highly susceptible to oxidative stress, so damage by porphyrin accumulation may explain the myelin deficit observed in our cases. In a mouse model for a severe form of porphyria, three oligodendrocyte proteins, MOG, PLP1 and CNP1, emerged through RNA sequencing of the hippocampus, indicating that oligodendrocytes are indeed involved in this group of disorders [2].

## Conclusions

We reviewed 16 cases and add 2 own cases which, in summary, leads to a total of 18 cases, of whom 17 have genetically been confirmed. We conclude that homozygous variegate porphyria has a complex clinical phenotype. Besides the cutaneous symptoms it comes along with developmental delay and neurologic symptoms. The disease frequently manifests in the first months of life. Therefore, we suggest that in children with developmental disorder of unknown cause, early childhood epilepsy and growth retardation, a detailed assessment of dermatological history and further diagnostic investigations are important. An abnormally photosensitive or fragile skin of the patient or a family member may indicate a genetic porphyria. Some types of porphyria, especially variegate porphyria, but also acute intermittent porphyria [15] and hereditary coproporphyria [27] with biallelic variants may include neurodevelopmental disorders. Accordingly, biochemical and, where appropriate, genetic examinations are recommended. Our cases together with the two cases in the literature presenting with MRI findings add biallelic variegate porphyria to the manifold causes of hypomyelinating disorders [30]. As the previously clinically established diagnosis of HVP is genetically combining homozygous and compound heterozygous cases, we propose, as done in this review, to use as a more precise definition the term “biallelic variegate porphyria”.

## Data Availability

All data generated or analysed during this study are included in this published article including all references of the reviewed literature.

## Abbreviations

PPOX: Protoporphyrinogen oxidase
HVP: Homozygous variegate porphyria
VP: Variate porphyria
MRI: Magnetic resonance imaging
RNA: Ribonucleic acid
DNA: Desoxyribonucleic acid
5-ALA: 5-Aminolevulinacid
T1w: T1 weighted image
T2w: T2 weighted image
IR: Inversion recovery
MOG: Myelin-oligodendrocyte glycoprotein
PLP1: Proteolipid protein
CNP1: Cyclic nucleotide phosphodiesterase
CPOX: Coproporphyrinogen oxidase
